# Neuralgia of the Testis

**Published:** 1881-06-20

**Authors:** Geo. Halsted Boyland

**Affiliations:** Late Surgeon French Army, etc.


### NEURALGIA OF THE TESTIS.

BY GEO. HALSTED BOYLAND, A. M., M. D., LATE SURGEON FRENCH ARMY, ETC.

The surgeon will, now and then, meet with a case that by careful diagnostic exclusion cannot be designated as either orchitis, epididymitis, or vaginalitis.

Neuralgia of the testis, in a strict sense, is characterized by irregular attacks of heavy, sticking, tearing, burning pains in the testicle; in one case that recently occurred in my practice, the symptoms stopped here, but in the more severe cases they are accompanied by nausea and vomiting, generally with spasmodic shortening of the cremaster, and consequent drawing up of the testicle, entirely independent of external influences. Nevertheless, there are cases in which, after oft repeated attacks, an extreme sensibility of the testis remains, so that palpitation calls forth a fresh attack. The severity of single attacks can attain such a pitch as to throw the patient into a state of violent excitation, and cause him to be covered with perspiration, to dance about, shrieking. Almost always, neuralgia of the testis affects only one side.

*Ætiology.*—This is a dark point. The spermatic nerves can be painfully excited at times from the periphery; at others, from the spinal marrow. In some instances, neuralgia of the testis has been produced by irradiation, during the passage of a renal calculus through the ureter, analogous to the cramp of the cremaster, more often observed in this condition. Such a neuralgia can seldom be traced to a chronic orchitis. The disturbance in the digestive organs, to which single attacks of the evil have been attributed, is probably only due to a similar cause, and to one at the same time remaining unknown.

*Treatment.*—From the foregoing it will be logical to deduce that our therapeusis cannot be what is technically called *rational.* In the irregular intermitting cases, good results have been obtained from the exhibition of quinine and Fowler's solution. In general, as in other neuralgias, quieting and strengthening medicines ought to be employed. The preparations of opium, hyoscyamus, aconite, and belladonna have been given inwardly and applied outwardly with doubtful results. I have found the following of great service :—

℞ Tinct. cannabis indica.......................... gtt. xl.
Potass. brom.................................. ʒ ij.
Aqua destil.................................. ℥ iv M.
SIG.—Tablespoonful every hour, until relieved.

Of course, the bowels must be kept open; for this, mild saline laxatives are best adapted. Dry heat, applied to the testicle on cloths, is a valuable adjuvant. The continued internal administration of iron is good treatment. So, also, with turpentine, especially in cases where it is undoubtedly a question of kidney trouble. The patience of the sufferer is often exhausted by the persistency of the evil, and he begs for castration. But this sacrifice of a healthy organ should be rejected, the more so, as the disease is always of a more or less constitutional nature; nevertheless, comparatively minor operations, such as ligature of the veins of the spermatic cord, and incision of the tunica albuginea, have been productive of remarkably favorable results.—*Am. Specialist.*

